# Functional effects of heavy metal exposures on N6-methyladenosine (m6A) methylation and other Epitranscriptomic modifications in the central nervous system

**DOI:** 10.1016/j.neuint.2025.106064

**Published:** 2025-10-02

**Authors:** Niraj Lodhi, Lauren Powell, Jay S. Schneider

**Affiliations:** Department of Pathology and Genomic Medicine, Thomas Jefferson University, Philadelphia, PA, 19107, USA

**Keywords:** Heavy metals, Lead (Pb), Arsenic (As), Cadmium (Cd), Cobalt (co), Manganese (Mn), N6-methyladenosine (m6A), Epitranscriptomics

## Abstract

Among different RNA methylations, N6-methyladenosine (m6A) is the most abundant in the brain and determines the fate of RNA through reversible processes using methyltransferases, demethylases, and methylbinding proteins. The reversibility of m6A is an emerging regulatory mechanism for gene expression, regulating many aspects of RNA metabolism and influencing learning and memory processes. Global m6A profiles are dynamically modified via the activity of various writers, readers, and erasers. However, m6A alterations from exposure to heavy metals, including the metals lead (Pb), cadmium (Cd), cobalt (Co), and manganese (Mn) and the metalloid arsenic (As), and the impact on brain function, are not fully understood. This paper reviews recent work that may begin to shed light on how heavy metal exposures may affect m6A methylation and how this might impact central nervous system functioning.

## 1. Introduction

Epitranscriptomics refers to the study of post-transcriptional RNA modifications, such as the methylation of adenosine and cytosine, that do not alter the ribonucleotide sequence but can affect RNA function, stability, and translation efficiency. This rapidly evolving field is providing important new insights into the direct modifications of RNA as a layer of control over RNA function related to the fine-tuning of transcriptomic responses critical for normal neurobehavioral function as well as responses to various stressors, such as environmental neurotoxicants (Cerneckis et al., 2024; Zhao et al., 2017). Epitranscriptomic modifications occur in all types of RNA and are controlled by the activities of various proteins and enzymes that serve as writers, readers, and erasers (Malovic et al., 2021). The writer, reader, and eraser regulators give the epitranscriptome its reversible and dynamic properties by actively depositing, recognizing, and removing modifications, respectively, depending upon cellular needs (Imbriano et al., 2023).

Writers are methyltransferase enzymes, such as methyltransferase-like 3 (METTL3) and methyltransferase-like 14 (METTL14), that deposit methyl groups on RNA bases after forming a complex with regulatory proteins like Wilms’ tumor 1-associating protein (WTAP) (Malovic et al., 2021). Additional proteins, including vir-like m6A methyltransferase associate (KIAA1429), RNA binding motif protein 15 (RBM15), and zinc finger CCCH-type containing 13 (ZC3H13), play vital roles in guiding, binding, and bridging the methyltransferase complex to specific RNA sites (Jiang et al., 2021). Erasers are demethylases, such as fat mass and obesity-associated alpha-ketoglutarate-dependent dioxygenase (FTO) and AlkB homolog 5 RNA demethylase (ALKBH5), that reverse methyltransferase activity by removing methyl groups from RNA bases (Jia et al., 2011; Zheng et al., 2013). Readers are RNA-binding proteins that identify and bind to modified RNAs and mediate downstream processes such as transcription, nuclear export, translation, alternative splicing, and decay (Imbriano et al., 2023; Leonetti et al., 2020). Examples of readers include those within the YT521-B homology domain family (YTHDF), including nuclear reader YTHDC1, cytoplasmic readers YTHDF1, YTHDF2, YTHDF3, and YTHDC2, and the insulin-like growth factor-2 binding proteins (IGFBP1-3) (Widagdo et al., 2022).

Each reader plays a specific role in downstream processing. For instance, YTHDC1 promotes splicing (Xiao et al., 2016) while YTHDF1 promotes translation (Shi et al., 2018; Wang et al., 2015), and YTHDF2 reduces stability (Shi et al., 2017) while IGF2BP1-3 enhances stability (Huang et al., 2018). Furthermore, YTHDC2 enhances translation (Hsu et al., 2017), and YTHDF3 mediates either translation or degradation depending upon whether it is interacting with YTHDF1 or YTHDF2, respectively (Shi et al., 2017). Readers play a key role in determining the fate of modified RNAs, affecting various biological processes (Chen et al., 2024). For example, Shi et al. (2018) reported that the reader binding protein YTHDF1 facilitates N6-methyladenosine (m6A) in promoting protein translation of targeted m6A-methylated mRNAs in mouse hippocampus in response to neuronal stimulation, and this process contributes to learning and memory (Fig. 1). Mice with genetic deletion of *Ythdf1* showed learning and memory deficits as well as impaired hippocampal synaptic transmission and long-term potentiation. Re-expression of YTHDF1 in the hippocampus of adult *Ythdf1*--knockout mice rescued the behavioral and synaptic defects, whereas hippocampus-specific acute knockdown of *Ythdf1* or *Mettl3* recapitulated the hippocampal deficiency. Thus, YTHDF1 appears to facilitate translation of m6A-methylated neuronal mRNAs in response to neuronal stimulation, which ultimately contributes to learning and memory (Shi et al., 2018).

The most extensively studied RNA modification is m6A, which is present transcriptome-wide in over a quarter of all RNAs and amounts to about half of all methylated ribonucleotides, making it the most abundant internal RNA modification (Zhang et al., 2019). It is typically located in a consensus motif (DRACH/GGACU) and enriched near stop codons and in 3′ UTRs (Dominissini et al., 2012; Linder et al., 2015; Meyer et al., 2012). The writer, reader, and eraser regulators were first identified in the m6A modification, which ultimately led to the discovery that RNA modifications are dynamic and reversible rather than static and unchangeable (Cao et al., 2016). The presence of specific writer, reader, and/or eraser regulators impacts how m6A modifies RNA, resulting in downstream effects on all aspects of RNA metabolism (Garbo et al., 2021; Zhao et al., 2017). Further, the regulators are linked to specific biological functions (Yao et al., 2024). For example, METTL3 plays an essential role in stem cell differentiation (Batista et al., 2014), YTHDF2 is crucial for the degradation of maternal mRNA and the subsequent activation of the zygotic genome (Deng et al., 2020), and FTO has been suggested to play an important role in the dopamine synaptic signaling pathway by increasing the synaptic dopamine concentration through its inhibition of the dopamine transporter (DAT) (Hess et al., 2013). Additionally, in the brain, m6A and in particular FTO, play a functional role in plasticity, learning, and memory by modifying the stability/decay of transcripts and the export, localization, and translation of mRNAs (Widagdo et al., 2022).

Alterations in the activity of various m6A regulators have been suggested to contribute to various neurological diseases and disorders (Yao et al., 2024). For example, alterations in m6A levels may play a role in the pathogenesis of Alzheimer’s disease (AD) (Xia et al., 2023). In patients with AD, the methyltransferase METTL3 has been found to be downregulated in post-mortem hippocampus and cortex, resulting in decreased m6A levels (Huang et al., 2020). Decreased m6A levels have also been observed in the middle temporal gyrus in patients with mild cognitive impairment (MCI), an early symptomatic stage that may precede AD (Zhao et al., 2021). In male and female wild-type C57BL/6 mice, *Mettl3* knockout in the hippocampus resulted in memory deficits and neuronal apoptosis, suggesting that METTL3 may be critical for hippocampal-dependent memory and could serve as a target for mitigating cognitive dysfunctions in AD (Zhao et al., 2021). Additionally, FTO expression was increased in 3xTg-AD mice, resulting in increased Tau protein phosphorylation, a biomarker for AD (Li et al., 2018).

A number of heavy metals are ubiquitous in the environment, and exposure to these environmental toxicants may increase the risk of developing various diseases and disorders that at least in part may be related to alterations in RNA modifications (Cayir et al., 2020). For instance, lead exposure may increase FTO expression and decrease METTL3 expression in cognition-related genes, resulting in impaired learning and memory in Sprague Dawley rats (Table 1) (Li et al., 2021). Cobalt exposure decreases FTO expression in apoptosis-related genes, resulting in abnormal apoptosis in human neuroglioma H4 cells (Tang et al., 2022). High-dose manganese exposure in adult C57BL/6 mice decreases FTO expression in ephrin-B2 RNA in the striatum, resulting in motor dysfunction and injury to dopaminergic neurons (Table 1) (Qi et al., 2022).

Heavy metals are defined as metal elements with high atomic weights and densities greater than 5 g/cm^3^; however, the term ’heavy metals’ is frequently used to describe elements that are toxic to the environment and biological organisms, which include certain metalloid elements in addition to metal elements (Briffa et al., 2020). Metalloid elements have properties that are intermediate between metals and nonmetals and can act as either a metal or nonmetal depending upon the reaction (Reichelt-Brushett and Batley, 2023). In this review, both toxic metals (i.e., lead, cadmium, cobalt, and manganese) and the metalloid element arsenic will be discussed. Lead, arsenic, and cadmium are among the most common heavy metal pollutants, while cobalt and manganese are essential trace elements that are toxic following excessive exposures (Briffa et al., 2020). While literature is abundant regarding the epitranscriptomic effects of these heavy metals and their implications in cancer, there is much less literature on the epitranscriptomic effects of heavy metal exposures on the central nervous system. To our knowledge, this is the first review of the effects of heavy metals on the epitranscriptome with a focus on effects on the nervous system.

## 2. Effect of heavy metals on m6A methylation

### 2.1. Lead (Pb)

It is estimated that about 800 million children around the world are exposed to lead from various sources (Rees and Fuller, 2020). In the US, millions of children are exposed to lead through water delivered via lead service lines, deteriorating lead-containing house paint, and lead-contaminated soil. Exposure to lead is particularly dangerous for children, in whom it can have significant negative impacts on neurodevelopment and cause functional changes resulting in adverse cognitive/behavioral outcomes that can persist into adulthood (Schneider, 2023).

Ai et al. (2023) reported MeRIP-seq and RNA-seq data from an *in vitro* study showing that lead exposure (5 μM lead acetate (PbAc) for 48 h) altered global m6A profiles in rat primary hippocampal neurons (Table 2) and identified the phosphoinositide-3-kinase protein kinase B (PI3K-AKT) signaling pathway as being regulated by m6A. Further, GO and KEGG analyses showed enrichment of differentially methylated genes, including cyclic AMP-responsive element-binding protein 3-like protein 2 (CREB3L2), heat shock protein 90 beta family member 1 (HSP90B1), and integrin subunit beta 5 (ITGB5), in the PI3K-AKT signaling pathway. Functionally, these genes are related to synaptic plasticity (CREB3L2) (Sampieri et al., 2021), neuronal response to injury (ITGB5) (Pajoohesh-Ganji et al., 2012), and intercellular signal transduction (HSP90B1) (Graustein et al., 2018). Further, following lead exposure, mRNA expression levels of *Creb3l2* were downregulated while m6A levels were upregulated, mRNA and m6A levels on *Itgb5* were both downregulated, and mRNA expression levels of *Hsp90b1* were upregulated while m6A levels were downregulated. Overall, global m6A levels were significantly increased along with mRNA and protein levels of METTL3 and FTO (Table 2) (Ai et al., 2023).

Li et al. (2021) showed that in lead-exposed adult male Sprague Dawley rats (0.2 % PbAc solution via oral gavage for 14 days), FTO expression in the hippocampus significantly increased, METTL3 expression significantly decreased, and the global methylation level of m6A significantly decreased, which was accompanied by impaired learning and memory in the Morris water maze (MWM) (Table 1). Additionally, a group of lead-exposed animals received folic acid treatment (0.4 mg/kg bodyweight via oral gavage) starting on day 7 of lead exposure to investigate potential neuroprotective effects. Folic acid is a micronutrient essential for cellular growth, regeneration, and gene expression regulation (Merrell and McMurry, 2025) and has been shown to decrease oxidative stress by inhibiting reactive oxygen species (ROS) production in the kidney of lead-exposed male Sprague Dawley rats (Li et al., 2022). Lead-exposed animals that received the folic acid intervention showed decreased hippocampal FTO levels and increased m6A levels compared to the lead-only group, although these results were not statistically significant. Behaviorally, the folic acid intervention group showed significantly improved learning and memory in the MWM compared to the lead-only group (Li et al., 2021). In this study, although there was no significant change in the expression level of METTL3 or FTO, the authors suggested that folic acid itself may increase m6A methylation. Folic acid acts as a coenzyme in the single-carbon metabolism pathway that produces S-adenosylmethionine (SAM), the primary methyl donor for methylation reactions in the central nervous system (CNS) (Menezo et al., 2022). When taken as a supplement, folic acid increases the availability of these methylated units, thus strengthening the RNA methylation pathway in the hippocampus (Choi et al., 2014). Aspects of the Li et al., (2021) study that limit the interpretation and generalizability of the data include: use of a rat strain (Sprague Dawley) that has been found to be relatively resistant to neurotoxic effects of lead (Verma and Schneider, 2017); use of adult animals only; acute (14 days) exposure to a high dose of lead (0.2 % PbAc solution, equivalent to approximately 1274 ppm); and no clarity as to when after lead exposure blood was sampled for lead levels. Further, alterations in FTO and METTL3 levels were analyzed indirectly by immunofluorescence in hippocampal sections.

The limited work on the effects of lead exposure on the epitranscriptome makes it impossible at this time to know what these effects really are and what their functional implications might be. The studies described above have several potential drawbacks that limit their interpretation. The Ai et al. (2023) study provides some potentially important insights into the effects of lead on epitranscriptomic machinery; however, the concentration of lead used for these *in vitro* studies on HT-22 cells (5 μM, equivalent to approximately 103.6 μg/dL) is very high and is not representative of a typical environmental exposure.

Additionally, the *in vitro* and *in vivo* studies by Ai et al. (2023) and Li et al. (2021), respectively, reported contradictory findings: Ai et al. (2023) reported an *increase* in global m6A levels while Li et al. (2021) reported a *decrease* in global m6A levels following lead exposure. The use of different species and differences in experimental conditions may explain the inconsistencies. Thus, additional studies are needed to gain insights into the effects of environmentally relevant lead exposures on the epitranscriptome during various periods of development, what the functional implications might be of any epitranscriptomic changes, and how this information may lead to new therapeutic targets for ameliorating the negative effects of lead exposure on the developing brain. Studies are also needed in female animals to determine if there are any sex-dependent effects of lead on the epitranscriptome.

### 2.2. Arsenic (As)

In its inorganic form, arsenic is highly toxic, and long-term exposure increases the risk of cognitive dysfunction, skin lesions, cardiovascular disease, diabetes, cancer, and death (Kuivenhoven and Mason, 2025). It is estimated that up to 220 million people worldwide are exposed to levels of arsenic higher than the WHO’s exposure limit of 10 g/L, primarily from contaminated groundwater (Podgorski and Berg, 2020) and arsenic-contaminated food items (Upadhyay et al., 2019). Arsenic exposure is particularly harmful to children. Children’s blood arsenic levels are negatively correlated with general intellectual ability, working memory, verbal comprehension, and processing speed, as measured by the Wechsler Intelligence Scale for Children – Fourth Edition (WIS-C-IV) (Wasserman et al., 2018).

Epitranscriptionally, arsenic (NaAsO_2_) administered via drinking water to adult male C57BL/6J mice for 6 months at concentrations of 0.5 mg/L, 5 mg/L, and 50 mg/L led to a dose-dependent decrease in the expression of the demethylase FTO in the cerebral cortex, resulting in increased global m6A levels and learning and memory impairments in the Morris water maze (MWM) and radial-arm maze, as well as increased anxiety-like behavior (Table 1) (Bai et al., 2018). The concentrations of arsenic used were environmentally relevant and consistent with levels of arsenic (i.e., 4.44 mg/L) found in contaminated groundwater (He and Charlet, 2013) and in polluted industrial areas (i.e., ≥5 mg/L) (Laskar et al., 2015). Bai et al. (2018) also exposed PC-12 Adh cells to environmentally relevant concentrations of arsenic (0.5, 1.5, and μM NaAsO_2_) for 24 h and found a dose-dependent decrease in FTO and increase in global m6A levels (Table 2), which was consistent with their *in vivo* findings.

Zhou et al. (2024) reported that exposure of human neuroblastoma SH-SY5Y cells to arsenic at environmentally relevant concentrations (1, 2.5, and 5 μM NaAsO_2_) resulted in a dose-dependent increase in oxidative stress, leading to a subsequent decrease in cell viability. These arsenic exposures increased global m6A levels in a dose-dependent manner in concert with the downregulation of FTO protein (Table 2). Arsenic exposure also resulted in significantly decreased mRNA and protein expression of activating transcription factor 3 (ATF3), which plays an important role in mediating oxidative stress responses, in a YTHDC1-mediated m6A-dependent manner through the downregulation of FTO. Overexpression of FTO protein significantly reduced arsenic-induced oxidative stress by decreasing mitochondrial superoxide anions. Additionally, arsenic-induced oxidative stress was exacerbated following *Fto* gene knockout. No significant arsenic-induced changes were observed in the protein expression of any methyltransferase or the demethylase ALKBH5 (Zhou et al., 2024).

Follow-up *in vivo* studies by Zhou et al. (2024) used adult male C57BL/6J wild-type, *Fto* knock-in, and *Fto* knockdown mice. Wild-type mice (groups included: control, 0.5 mg/L, 5 mg/L, and 10 mg/L NaAsO_2_) exhibited increased oxidative stress and a dose-dependent increase in m6A following exposure to arsenic via drinking water for 3 months (Table 1). A significant decrease in FTO and increase in WTAP protein expression in the cortex following 10 mg/L NaAsO_2_ exposure over 3 months was also seen (Table 1). Behaviorally, wild-type mice showed spatial learning and memory deficits and short-term working memory impairments following exposure to arsenic at concentrations greater than 5 mg/L (Table 1). *Fto* knock-in mice exposed to 10 mg/L NaAsO_2_ via drinking water showed reduced oxidative stress, determined by decreased expression of heme oxygenase-1 (HO-1), glutamate-cysteine ligase (GCLM), and gamma-H2A histone family member X (γ-H2AX), as well as reduced m6A levels in the cortex, compared to wild-type mice exposed to arsenic. In contrast, *Fto* knockdown mice exposed to 10 mg/L NaAsO_2_ had increased oxidative stress and increased m6A levels in the cortex. Behaviorally, upregulation of FTO reduced short-term working memory impairments seen in arsenic-exposed wild-type mice, while *Fto* knockdown mice exhibited worse learning and memory impairments, in the MWM and Y-maze tests, following arsenic exposure. Finally, *Fto* knock-in mice showed reduced neuron loss in the cortex and hippocampus following arsenic exposure, while *Fto* knockdown mice showed increased neuron loss following arsenic exposure, compared to arsenic-exposed wild-type mice (Zhou et al., 2024). Overall, these studies suggest that upregulation of FTO may play a neuroprotective role by decreasing oxidative stress following arsenic exposure.

Together, these data suggest that arsenic exposure may result in cognitive dysfunction, at least in part mediated by epitranscriptomic modulation. Additional studies are necessary to elucidate the exact molecular mechanisms, though current studies suggest that FTO is a key regulator in arsenic-induced m6A modification. Current studies have mainly focused on oxidative stress reactions, so additional studies are necessary to elucidate other biological processes that may be affected by arsenic exposure. Additionally, information obtained by exposure via drinking water may not accurately reflect the nature of arsenic-induced epitranscriptomic changes that occur following exposure via food due to absorption differences. Further, studies to date have used only adult mice despite the high susceptibility of children to arsenic toxicity (Tyler and Allan, 2014), thus, developmental exposure studies are needed to understand the effects of arsenic exposure on the epitranscriptome in children. Finally, the relevance of these data should be carefully considered prior to applying any findings to females, as only male mice have been used and sex-specific differences may exist.

### 2.3. Cadmium (Cd)

Cadmium poses significant environmental and public health concerns due to its high toxicity and persistence in ecosystems (Charkiewicz et al., 2023). Cadmium exposure through air should be no greater than 5 ng/m^3^ while exposure through drinking water should be limited to 0.003 mg/L (Briffa et al., 2020). Exposure to cadmium occurs primarily through dietary intake (soil-to-plant transfer and contamination of animal products), inhalation of cigarette smoke, and occupational exposure in industries such as mining, metal refining, battery manufacturing, and waste incineration (Satarug et al., 2010; Wang et al., 2021). Cigarette smokers have an increased risk for cadmium toxicity compared to non-smokers, as one cigarette can result in inhalation of approximately 0.1–0.2 μg of cadmium, which is greater than the WHO’s air quality exposure guidelines (Ashraf, 2012). Additionally, according to the Agency for Toxic Substances and Disease Registry, over 500,000 workers are exposed to cadmium levels greater than OSHA’s legal limit of 5 μg/m^3^ over an 8-h workday (Jaishankar et al., 2014).

Epitranscriptionally, cadmium exposure may increase global m6A levels by increasing the mRNA and protein expression of the m6A methyltransferases METTL3 and METTL14, as observed in male C57BL/6 mouse hippocampal tissues and mouse neuroblastoma cells, respectively (Tables 1 and 2) (Deng et al., 2023; Zhang et al., 2024). Studies have also found that the YTHDF2 reader protein that promotes mRNA degradation may also increase following exposure to 10 μM CdCl_2_ in HT-22 cells (derived from mouse hippocampal neurons) (Table 2) (Zhang et al., 2024). Long-term CdCl_2_ exposure (72 h; 1, 2, and 4 μM) in mouse neuroblastoma (Neuro-2a) cells and mouse hippocampal neurons (0.5, 1, and 1.5 μM) in culture significantly decreased mitochondrial membrane potential and ATP levels and increased mitochondrial reactive oxygen species (ROS) production in a concentration-dependent manner, resulting in cytotoxicity (Deng et al., 2023). These were relatively low-level exposures compared to the WHO and OSHA acceptable exposure limits. Additionally, lnc-*Gm10532* expression levels were significantly increased following chronic CdCl_2_ exposure, and knockdown of lnc-*Gm10532* using a small interfering RNA reversed cadmium’s negative effects on the mitochondrial membrane potential, ATP levels, and mitochondrial ROS production (Deng et al., 2023). Downstream targets of lnc-*Gm10532*, including mitochondrial fission 1 protein (FIS1) and sequestosome 1 (SQSTM1), most likely mediated these effects (Deng et al., 2023). Chronic CdCl_2_ exposure also increased protein expression of METTL14, resulting in increased m6A levels on *Fis1* mRNA (Table 2) (Deng et al., 2023). Knockdown of lnc-*Gm10532* antagonized increased m6A levels; however, overexpression of METTL14 partially reduced this antagonization and resulted in increased mRNA levels of *Fis1*, suggesting that lnc-*Gm10532* recruits METTL14, increases m6A levels, and ultimately stabilizes *Fis1* mRNA (Deng et al., 2023).

Male C57BL/6J mice exposed to environmentally relevant levels of cadmium (0.6 mg/L CdCl_2_, within the range of blood cadmium levels found within US non-smokers (Wang et al., 2020), via drinking water for 6 months showed spatial learning and memory impairments (Deng et al., 2023). Histopathologically, nuclear shrinkage and neuronal cell loss were observed in the hippocampal regions CA3 and dentate gyrus (DG), and RT-qPCR showed significantly increased lnc-*Gm10532* and *Fis1* expression levels in the hippocampus (Deng et al., 2023). Overall, the results suggest that increased lnc-*Gm10532* expression may be negatively correlated with spatial learning and memory and that chronic cadmium neurotoxicity may be regulated by lnc-*Gm10532*-FIS1 signaling, which is modulated epitranscriptionally (Deng et al., 2023).

In other studies, the effects and mechanisms of adolescent exposure to cadmium and a high-fat diet (HFD) on behavioral and histological outcomes were examined in male C57BL/6 mice (Zhang et al., 2024). Mice exposed to 100 mg/L CdCl_2_ (a concentration that results in serum cadmium levels (7.21 ± 0.74 μg/L) that are similar to levels found in cognitively impaired patients exposed to cadmium through industrial pollution and/or dietary intake (Iqbal et al., 2018)) via drinking water for 10 weeks showed impaired spatial learning and memory in the Morris water maze (MWM) (Table 1). Co-exposure to CdCl_2_ and HFD (60 % fat) (HCd) resulted in a significant increase in anxiety-like behavior in the elevated plus maze and open field test compared to both control and CdCl_2_-only exposed groups (Zhang et al., 2024). Additionally, the HCd group had significant impairments in spatial learning and memory compared to control and CdCl_2_-only groups, indicating that co-exposure to CdCl_2_ and HFD exacerbated the negative effects of CdCl_2_ exposure on spatial learning and memory (Zhang et al., 2024). Histologically, CdCl_2_ exposure resulted in decreased dendritic spine densities on hippocampal CA1, CA3, and DG neurons and decreased expression of Synapsin-1, post-synaptic density protein 95 (PSD95) and brain-derived neurotrophic factor (BDNF) in the hippocampus, which was further exacerbated following HCd exposure (Zhang et al., 2024).

Cadmium chloride exposure increased methyltransferase METTL3 mRNA and protein levels, resulting in an increase in global m6A levels in the hippocampus (Table 1) (Zhang et al., 2024). Hippocampal YTHDF2 mRNA and protein levels were also significantly increased following CdCl_2_ exposure (Table 1). The increase in global m6A levels, METTL3, and YTHDF2 was exacerbated in the HCd mice, which induced La ribonucleoprotein 7 (LARP7) mRNA degradation and a reduction in the interaction between LARP7 and Sirtuin 6 (SIRT6) (Zhang et al., 2024). Consequently, SIRT6 protein expression was decreased and its deacetylase activity inhibited, resulting in increased hippocampal senescence, which may play a role in the cognitive dysfunction in the HCd mice (Zhang et al., 2024). Exposure of HT-22 cells to 10 μM CdCl_2_ and a combination of 10 μM CdCl_2_ and 200 μM of the main component of dietary saturated fat, palmitic acid, also significantly increased YTHDF2 protein and mRNA levels. Overall, the studies by Zhang et al. (2024) suggest that both CdCl_2_ exposure alone and in combination with HFD may increase the protein and mRNA expression of the methyltransferase METTL3 and the reader YTHDF2, resulting in increased global m6A levels on *Larp7* mRNA and increased degradation of *Larp7* mRNA in mouse hippocampal neuronal cells.

Together, these data suggest that cadmium exposure may increase oxidative stress and neuronal senescence and result in cognitive dysfunction at least in part by increasing global m6A levels, although further studies are necessary to reveal the exact molecular mechanisms underpinning these processes. Although the results of studies looking at serum cadmium levels corresponding to the levels found in patients with cognitive impairment may be beneficial in elucidating processes involved in cadmium-induced cognitive impairment, the relevance of these data should be carefully considered before applying any findings to cadmium-induced changes that occur following typical environmental exposures that result in lower serum cadmium levels. Additionally, biological processes other than those involved in oxidative stress and neuronal senescence may be regulated following cadmium exposure; thus, further investigation is necessary. Future studies should also include females, as sex-specific differences may exist. Lastly, cadmium exposure primarily occurs through inhalation; therefore, information obtained by exposure through drinking water may not reflect the nature of cadmium-induced epitranscriptomic changes that occur through inhalation.

### 2.4. Cobalt (co)

Cobalt is extensively used in aerospace applications due to its strength, stability, and corrosion resistance, and is found in ceramic pigments, lithium-ion batteries, and in artificial alloy limb prostheses (Kang et al., 2013). Blood cobalt levels of at least 2.5 μg/L (less than the assumed safety threshold of 10 μg/L) have been associated with neurodegenerative changes in the brain (Tang et al., 2023). Cobalt exposure has also been associated with impaired memory and sensory functions and may be a risk factor for the development of Alzheimer’s disease (AD) (Tang et al., 2020, 2023; Zheng et al., 2023).

Cobalt-induced ferroptosis may lead to neurodegeneration through epigenetic regulation of ALKBH5 (Su et al., 2024; Zheng et al., 2023). Ferroptosis is a mechanism for non-apoptotic, iron-dependent, oxidative cell death that is characterized by glutathione consumption and lipid peroxide accumulation (Bao et al., 2021; Hirschhorn and Stockwell, 2019; Petrova et al., 2020). Heme oxygenase-1 (HO-1) is a direct target gene of ALKBH5, and both cobalt exposure (CoCl_2_; 400 μM) and *Alkbh5* knockdown of H4 (human neuroglioma) cells decreased ALKBH5 expression and upregulated m6A modification of *Ho*-1 mRNA (Table 2) (Su et al., 2024). Cobalt exposure (4, 8, and 12 mg CoCl_2_/kg bwt by intraperitoneal injection daily for 30 days) of 10-week-old male C57BL/6 mice increased HO-1 protein levels in the cortex in a concentration-dependent manner (Su et al., 2024). ALKBH5 may directly regulate the m6A modification level of *Ho-*1 mRNA, thereby influencing its post-transcriptional processing (Su et al., 2024; Zheng et al., 2020). Thus, enhancing ALKBH5 expression may potentially play an important role in mitigating cobalt-induced ferroptosis.

Adult C57BL/6 male mice exposed to either 8 or 16 mg CoCl_2_/kg bwt by intraperitoneal injection once a day for 30 days developed cortical neuronal cell death and neurofibrillary tangles in the cerebral cortex (Table 1) (Tang et al., 2020). Intraperitoneal injection mimics aspects of cobalt exposure that may arise from implant deterioration experienced by those with metal-on-metal (MOM) hip joint replacements, and the doses given were in the range of cobalt blood levels found in patients with MOM hip joint replacements (Gómez-Arnaiz et al., 2022). Behaviorally, CoCl_2_ exposure impaired spatial learning and memory in the Morris water maze (MWM) and, epitranscriptionally, significantly decreased global m6A levels in the cortex (Table 1) (Tang et al., 2020). Cobalt-induced alterations in m6A levels were found on 730 neurodegeneration-associated genes, including some that have been implicated in Alzheimer’s disease and Huntington’s disease. Cobalt chloride exposure (16 mg/kg bwt) also resulted in a significant increase of FTO protein and significantly decreased METTL3, METTL14, and WTAP protein levels in the cortex (Table 1) (Tang et al., 2020). There was no significant change in ALKBH5 protein levels, but there was a significant increase in *Alkbh5* mRNA expression in the cortex following 16 mg CoCl_2_/kg bwt exposure (Tang et al., 2020).

Tang et al. (2023) also reported that exposure of human neuroglioma H4 cells to a high concentration (400 μM) of CoCl_2_ (that may not be relevant to environmental exposures) for 24 h increased m6A levels in concert with decreased FTO levels (Table 2) and increased protein levels of the autophagy marker microtubule-associated protein 1 light chain 3B-II (LC3B-II), glycogen synthase kinase 3 beta (GSKβ), and phosphorylated Tau at Threonine 181 and Serine 262 sites. Overexpression of FTO decreased global m6A levels, LC3B-II protein expression, and autophagosome accumulation, as well as reversed decreased lysosomal-associated membrane protein 2 (LAMP2) expression and partially attenuated increased p-Tau protein expression at Thr181 and Ser262 that was induced by CoCl_2_ exposure. MeRIP-seq revealed 42 differentially expressed m6A-modified genes implicated in neurodegenerative diseases, such as AD, following CoCl_2_ exposure. FTO downregulation resulting from CoCl_2_ exposure also increased tuberous sclerosis complex 1 (TSC1) mRNA expression, and *Tsc1* siRNA reversed increased LC3B-II and p-Tau protein levels following CoCl_2_ exposure, suggesting that TSC1 may be a target for cobalt-induced autophagy and neurodegeneration. Further, CoCl_2_ exposure resulted in decreased YTHDF2 protein expression (Table 2), and YTHDF2 overexpression significantly decreased *Tsc1* mRNA stability, suggesting that FTO may regulate *Tsc1* mRNA stability in an m6A-YTHDF2 manner (Tang et al., 2023).

Studies with 8-week-old male Nestin-cre mice showed that *Fto* conditional knockout (cKO), CoCl_2_ exposure (intraperitoneal injection of 12 mg/kg bwt once daily for 30 days, resulting in blood cobalt levels in the range found in patients with MOM implants), and cKO + CoCl_2_ all resulted in significantly impaired learning and memory in the MWM, with the cKO + CoCl_2_ mice having the greatest deficits (Tang et al., 2023). All three groups of mice had increased TSC1 expression and increased p-Tau (Thr181) in the hippocampus (DG, CA1, and CA3) compared to control mice, with the cKO + CoCl_2_ mice having the greatest increase in p-Tau (Thr181) (Tang et al., 2023). Several key proteins involved in regulating synaptic plasticity, including N-methyl-d-aspartate receptor 1 (NMDAR1), NMDAR2A, and NMDAR2B, were significantly decreased in the hippocampus in the cKO mice (Tang et al., 2023). Interestingly, there were fewer changes (compared to the cKO mice) in NMDA receptors following CoCl_2_ exposure alone and in combination with cKO, where CoCl_2_-exposed mice only had significantly decreased NMDAR2A protein levels, and cKO + CoCl_2_ mice only had significantly decreased NMDAR2B protein levels (Tang et al., 2023). Further studies are necessary to more fully understand the effects of cobalt exposure on the nervous system and the role that FTO may play in the expression of NMDA receptors.

Blood samples from patients with MOM hip replacements showed significantly increased cobalt concentrations and significantly decreased FTO and ALKBH5 expression (Tang et al., 2023; Zheng et al., 2023). In H4 and SHSY5Y cell lines, exposure to CoCl_2_ (400 and 600 μM in H4 and 200 and 400 μM in SHSY5Y cells) for 24 h resulted in significantly decreased ALKBH5 protein expression (Zheng et al., 2023). MeRIP-seq of *Alkbh5* knockdown and CoCl_2_-exposed H4 cells showed overlapping differentially m6A-modified genes enriched in neurodegenerative disease pathways (Zheng et al., 2023). Adult male C57BL/6 wildtype mice and *Alkbh5* knockout mice administered 16 mg CoCl_2_/kg bwt (the higher range of cobalt levels found in hip replacement patients) by intraperitoneal injection once a day for 30 days showed increased neurofibrillary tangles and expression of p-Tau in the hippocampus. The expression of the Alzheimer’s-related proteins amyloid precursor protein (APP) and p-Tau significantly increased following cobalt exposure, with *Alkbh5* knockout exacerbating the increase in APP (Zheng et al., 2023).

Together, these data may suggest a potentially increased risk of developing AD following cobalt exposure that is at least in part driven by the effects of cobalt on the epitranscriptome. Additional studies, however, are needed to further elucidate the mechanisms of cobalt-induced m6A modifications. Although current data from patients with MOM hip replacements suggest that cobalt exposure decreases expression of the m6A demethylases FTO and ALKBH5, Tang et al. (2020) reported an *increase* in the demethylase FTO following CoCl_2_ exposure in mice, while *in vitro* studies (Tang et al., 2020, 2023) reported a *decrease* in FTO expression following CoCl_2_ exposure. Considering these inconsistent findings, further studies are necessary to provide clarity regarding cobalt effects on epitranscriptomic modulation, although species diversity and differences in experimental conditions may explain the inconsistencies, as fewer genes were changed/upregulated *in vitro* versus *in vivo* (Tang et al., 2020). Further, the concentration of CoCl_2_ used in H4 cell studies was specifically used to study the mechanisms of toxicity following excessive cobalt exposure and was not environmentally relevant; thus, any findings should be carefully considered in view of the high exposure compared to typical environmental exposures. Lastly, studies have used only male mice, so additional studies using females are necessary to determine whether any sex-specific differences may exist.

### 2.5. Manganese (Mn)

Manganese is an essential trace metal and micronutrient that plays a role in a variety of neurological functions (Parsons-White and Spitzer, 2018). However, exposure to high levels of manganese can lead to a variety of neurological disorders, including manganese-induced Parkinsonism, with cognitive dysfunction and motor impairments that resemble those seen in patients with Parkinson’s disease (PD) (Martins et al., 2020; Peres et al., 2016).

Wen et al. (2024) exposed male C57BL/6 mice to different concentrations of MnCl_2_ (14, 28, and 56 mg/kg bwt) via oral gavage for 56 days and reported a dose-dependent downregulation of FTO mRNA and protein levels and increased global m6A levels in the hippocampus that were associated with learning and memory impairments in the Morris water maze and Novel Object Recognition Test (Table 1). Adenoviral vector and valproic acid (VPA) (4 mg/kg bwt, subcutaneous (s.c.))-induced overexpression of FTO and MA2 (20 mg/kg bwt, s.c.)-induced inhibition of FTO were associated with reducing and worsening, respectively, manganese-induced learning and memory impairments. Further, RNA-seq on HT-22 cells (an immortalized mouse hippocampal cell line) overexpressing FTO and exposed to manganese found that manganese exposure significantly altered expression of several GRIN subtype NMDA receptors and that manganese-induced downregulated FTO expression (in concert with increased YTHDF3 expression) increased m6A modification levels (Table 2) in several glutamate receptor, ionotropic, N-methyl d-aspartate (GRIN) transcripts (i.e., GRIN1 and GRIN3B) leading to their accelerated degradation. Further, studies of manganese-mediated transcriptional regulation of FTO revealed that manganese downregulated transcription factor sex determining region Y-box 2 (SOX2), which in turn inhibited FTO transcription (Wen et al., 2024).

Qi et al. (2022) examined the role of m6A and FTO in manganese-induced motor dysfunction in mice (C57BL/6, age and sex not specified). Some animals were given daily injections of 12.5, 25, or 50 mg MnCl_2_/kg bwt via intraperitoneal injection for two weeks. Other animals received striatal injections of AAV5-FTO, AAV5-ephrin-B2 (ephrin-2 plays an important role in the development and functioning of the dopamine system), or AAV5 silencing METTL3 (METTL3si), which were then all followed by 50 mg MnCl_2_/kg bwt exposure as described above. Manganese exposure decreased striatal dopamine levels and neuronal axon length, and these effects were partially protected in AAV5-FTO animals. The mRNA and protein levels of FTO were also significantly downregulated in the striatum after manganese exposure; however, levels of METTL3/14, ALKBH5, and YTHDF1/2 were not significantly affected by manganese exposure. Manganese exposure led to a variety of motor impairments that were also reduced in AAV5-FTO and METTL3si animals. Further, manganese-induced downregulation of FTO in SH-SY5Y cells enhanced mRNA m6A modification of ephrin-B2 (Table 2), which, when recognized by YTHDF2, is degraded. Thus, regulation of ephrin-B2 appears to be at least partly regulated through an m6A/YTHDF2 mechanism. These authors went on to further suggest that perhaps manganese-induced Parkinsonism is characterized by an increase in striatal m6A levels that may lead to degradation of ephrin-B2 mRNA through a YTHDF2-dependent mechanism (Qi et al., 2022). Additional studies are needed to determine if such manganese-induced epitranscriptomic changes are indeed associated with manganese-induced Parkinsonism.

Overall, the existing research suggests that m6A and FTO may play important roles in the development and expression of manganese-induced cognitive and motor dysfunction. Further studies are required to better understand the precise mechanisms involved in environmentally relevant manganese exposure effects of the epitranscriptome, and females need to be assessed to determine whether sex-specific differences in the epitranscriptomic response to manganese exist.

## 3. Conclusions and future directions

This review summarizes the relatively small body of literature on epitranscriptomic alterations in the nervous system induced by heavy metal exposures. Recent studies have provided evidence that exposure to heavy metals can alter global m6A profiles by regulating the expression of writers, erasers, and/or readers. However, further research is necessary to address various limitations in the literature. Primarily, additional studies should investigate the contradictory findings that have been reported across experimental conditions (*in vitro* vs. *in vivo*), particularly following lead and cobalt exposures. Although species diversity and differences in experimental conditions (i.e., the use of doses *in vitro* that exceed environmental relevance) may explain the inconsistencies, further studies using environmentally relevant concentrations are pertinent to provide clarity. Though using high concentrations *in vitro* provides insights into the mechanisms of toxicity following excessive heavy metal exposures, the findings may not translate to accurately reflect the effects of typical environmental exposures. Additionally, *in vivo* experiments would benefit from including both males and females to examine any potential sex-related differences in epitranscriptomic responses to heavy metal exposures. Currently, the effects of heavy metal exposures have been investigated only in males, which is a limitation of the current literature.

Although necessary during initial investigations, examining the epitranscriptomic effects of exposure to a single metal at a time is another limitation of the current literature, as co-exposure to various heavy metals is common in certain occupational settings and/or from environmental sources (Tchounwou et al., 2012). Thus, future studies might also include investigating the epitranscriptomic effects of combined exposures to various metals and metalloids. For instance, occupations such as construction and mining increase workers’ risks of co-exposures to lead, cadmium, and arsenic (Ceballos et al., 2022; Wongsasuluk et al., 2021), and environmental pollution increases the risk of co-exposures to various heavy metals, including cadmium, lead, manganese, chromium, and cobalt, from contaminated soil, water, and air (Mitra et al., 2022). Future studies might also include the study of epitranscriptomic alterations induced by early life (pre- or postnatal) heavy metal exposures and the extent to which these changes persist through the lifespan or are subject to transgenerational transmission. Children are highly susceptible to heavy metal toxicity throughout development, but the dynamic and reversible nature of RNA modifications may allow for the mitigation of certain adverse effects with further investigation. Lastly, future studies might include the examination of potential crosstalk between heavy metal-induced epigenetic modifications and epitranscriptomic alterations. Overall, the study of epitranscriptomic effects of heavy metal exposures and their functional significance is an exciting emerging area of investigation. We encourage researchers to embrace this new area of epitranscriptomics in the hope that it will provide new insights into heavy metal neurotoxicity and the role that these exposures may play in the development of chronic neurological diseases.

## Figures and Tables

**Figure:**
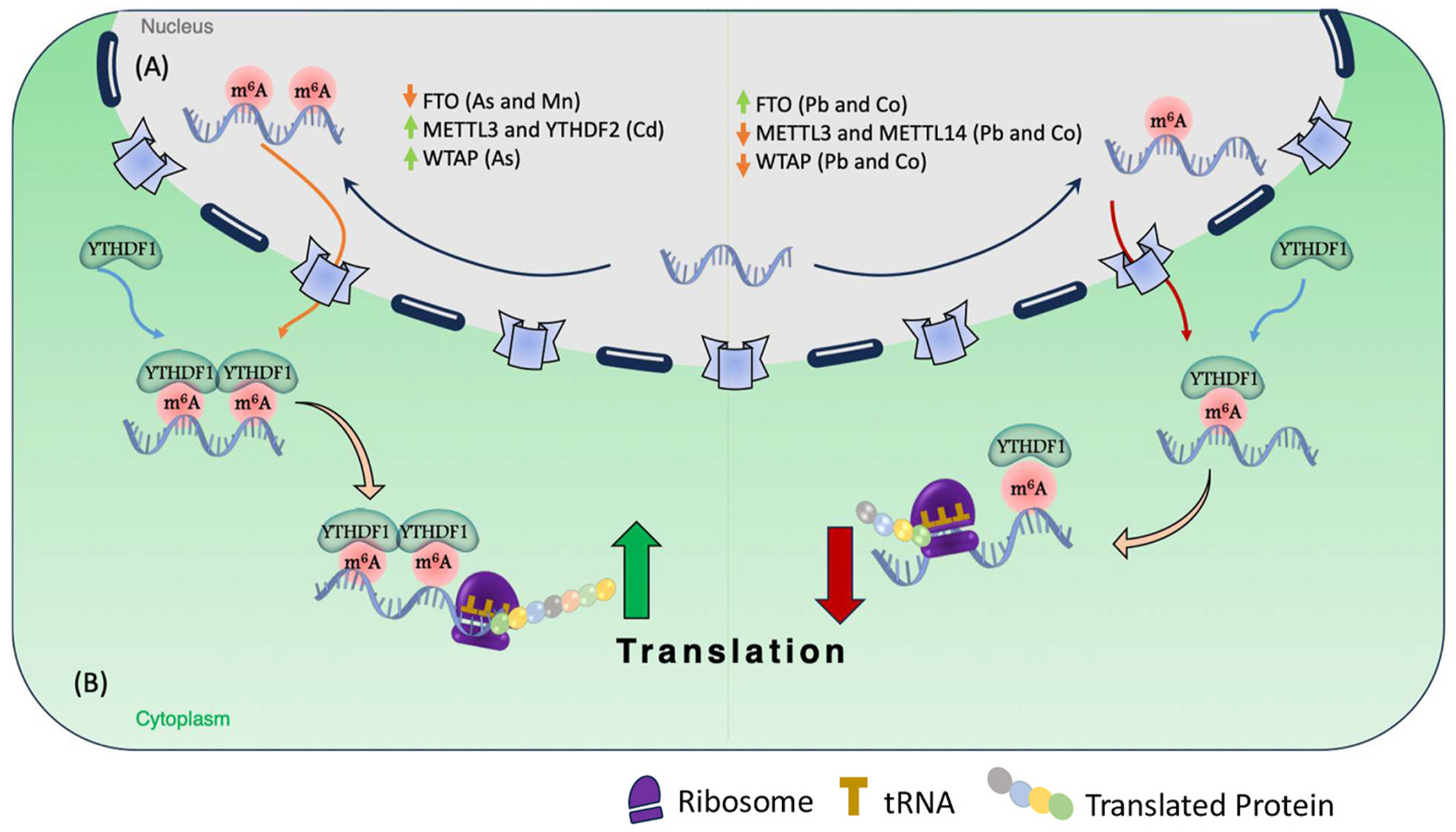
Overview of modifications of m6A by heavy metal exposures. Heavy metal exposure affects the m6A modification by differentially altering the expression of m6A writers such as METTL3, METTL14, WTAP, and readers and erasers, including YTHDF2 and FTO. In the cytoplasm, YTHDF1 selectively recognizes m6A-modified mRNAs, promoting protein synthesis of target transcripts by facilitating their recruitment to the ribosomal translational machinery and increasing the translation rate of methylated mRNAs. In addition, cadmium exposure also specifically increases the expression of YTHDF2, which in turn degrades targeted *Larp7* mRNA (not shown in the Figure), representing a key molecular mechanism linking and environmental exposure to potential neurodegenerative processes and cognitive deficits*.*

**Table 1:** Epitranscriptomic Effects of Heavy Metal Exposures *in vivo.*

	Metal	Species	Exposure Level	m6A Level	Expression of readers, writers and erasers	Effects on CNS/Functions	References
**1.**	**Pb**	**Male Sprague Dawley** (**SD) rats, aged 6 weeks.**	0.2 % PbAc in water via oral gavage for 14 days.	Decrease	FTO Up; METTL3 Down	Impaired learning, memory, and spatial localization.	Li et al. (2021)
**2.**	**As**	**Male C57BL/6J mice, aged 8 weeks.** **Male C57BL/6J mice, aged 8 weeks.**	NaAsO_2_ (0.5, 5, and 50 mg/L) via drinking water for 6 months. NaAsO2 (0.5, 5, 10 mg/L) via drinking water for 3 months.	Increase Increase	FTO Down FTO Down; WTAP Up	Impaired conditioned avoidance and escape responses; increased anxiety-like behavior. Increased oxidative stress; spatial learning and memory deficits; impaired short-term memory.	Bai et al., 2018 Zhou et al. (2024)
**3.**	**Cd**	**Male C57BL/6 mice, aged 5 weeks.**	CdCl_2_ (100 mg/L) via drinking water for 10 weeks.	Increase	METTL3 Up; YTHDF2 Up	Hippocampal neuronal senescence and damage; impaired spatial learning and memory.	Zhang et al. (2024)
**4.**	**Co**	**Male C57BL/6 mice, aged 8 weeks.**	CoCl_2_ (16 mg/kg bwt) IP daily for 30 days.	Decrease	FTO Up; METTL3, METTL14, and WTAP Down	Impaired spatial learning and memory; impaired synaptic transmission in cortex.	Tang et al. (2020)
**5.**	**Mn**	**Male C57BL/6 mice, aged 12 weeks.**	MnCl_2_ (28 and 56 mg/kg bwt) daily via oral gavage for 56 days.	Increase	FTO Down	Impaired hippocampal synaptic plasticity; impaired learning and memory.	Wen et al. (2024)

**Table 2:** Epitranscriptomid effects of heavy metal exposures *In Vitro*.

	Metal	Cell Type	Exposure Level	m6A Level	Expression of readers, writers and erasers	References
**1.**	**Pb**	**HT-22 cells, derived from P0 Sprague Dawley (SD) rat primary hippocampal neurons.**	PbAc (5 μM) for 48 h.	Increase	METTL3 Up; FTO Up	Ai et al. (2023)
**2.**	**As**	**PC-12 Adh cells, derived from rat adrenal gland tissue.** **SH-SY5Y cells, derived from human neuroblastoma.**	NaAsO_2_ (1.5 and 2.5 μM) for 24 h. NaAsO_2_ (1, 2.5, and 5 μM) for 24 h.	Increase Increase	FTO Down FTO Down	Bai et al., 2018 Zhou et al. (2024)
**3.**	**Cd**	**Neuro-2a cells, derived from mouse neuroblastoma.**	CdCl_2_ (4 μM) for 72 h.	Increase	METTL14 Up	Deng et al. (2023)
**4.**	**Co**	**H4 cells, derived from human neuroglioma.** **H4 cells, derived from human neuroglioma.**	CoCl_2_ (400 μM) for 48 h. CoCl_2_ (400 μM) for 24 h.	Increase Increase	ALKBH5 Down FTO Down; YTHDF2 Down	Su et al. (2024) Tang et al. (2023)
**5.**	**Mn**	**HT-22 cells, derived from mouse primary hippocampal neurons.** **SH-SY5Y cells, derivation not specified.**	MnCl_2_ (500 μM) for 24 h. MnCl_2_ (500 μM) for 24 h.	Increase Increase	FTO Down FTO Down	Wen et al. (2024) Qi et al. (2022))
